# The Nintendo^®^ Wii Fit Balance Board can be used as a portable and low-cost posturography system with good agreement compared to established systems

**DOI:** 10.1186/s40001-020-00445-y

**Published:** 2020-09-24

**Authors:** Ben Rohof, Marcel Betsch, Björn Rath, Markus Tingart, Valentin Quack

**Affiliations:** 1 Department of Orthopaedic Surgery, University Hospital RWTH Aachen, Pauwelsstr. 30, 52074 Aachen, Germany; 2 Department of Orthopaedic Surgery, Hospital Wels-Grieskirchen, Grieskirchner Str. 42, 4600 Wels, Austria

**Keywords:** Nintendo^®^ Wii Fit Balance Board, Screening, Postural stability, Fragility fractures, Geriatric assessment

## Abstract

**Background:**

Almost all epidemiological studies over the past 40 years have determined that the incidence of fragility fractures is increasing. Therefore, the assessment of postural stability and monitoring any progress during balance training for geriatric patients to prevent falls are becoming more important. The Nintendo^®^ Wii Fit Balance Board, with its integrated software and scoring system, might be a cheap and easily accessible tool for this purpose.

**Methods:**

This prospective study analyzed the diagnostic value of the Wii Fit Balance Board in 41 healthy subjects using two measurements: the yoga task “tree,” which is performed in one-leg stance; and the balance game “table tilt.” Our investigation compared these tasks to two established, regularly used systems, the MFT-S3 Check and the Posturomed, by looking for correlation and agreement, using Bland–Altman plots, as well as for differences to demographic data. All measurement tools were also compared to the Sensory Organization Test—the gold standard for detecting impaired balance.

**Results:**

We found a moderate correlation between the yoga exercise “tree” and the Sensory Organization Test (correlation coefficient *r* = 0.514, *p* = 0.001) as well as the MFT-S3 Check (*r* = 0.356–0.472, *p* = 0.002–0.022) and the Posturomed (*r* = 0.345, *p* = 0.027). However, results from the balance game “table tilt” did not show a significant correlation with those of the systems to which we compared it (*p* = 0.301–0.953).

**Conclusions:**

According to the literature, the raw data from the Wii Fit Balance Board are comparable to that obtained by laboratory-grade force platforms. We have found, however, that the yoga pose “tree,” as integrated into the Nintendo^®^ Wii Fit Balance Board with its own scoring system, also correlates with the gold-standard Sensory Organization Test. It also correlates with two frequently used diagnostic and therapeutic devices. We, therefore, conclude that the Wii Fit Balance Board is suitable for the evaluation of postural stability and may be useful in preventing falls among the geriatric population.

**Level of evidence:**

2b.

## Background

With demographic changes leading to an increase in fragility fractures, the need for postural stability training and testing is becoming more important medically, in particular among trauma and orthopedic surgeons. In 2005, there were nearly 2 million fragility fractures in the United States alone, a number expected to rise to more than 3 million by 2025 [1].

Fragility fractures are associated with major morbidities, loss of independence and increased mortality [2, 3] and lead to a moderate-to-high-risk of further falls [4]. Restoring mobility is, therefore, crucial to enabling elderly people to walk around freely, preserve autonomy and maintain social engagement.

Rubenstein et al. identified gait or balance disorders as a major cause of falls in elderly adults [5]; Ganz et al. reported that the most consistent predictors of future falls, with a likelihood ratio range of 1.7–2.4, were pre-existing gait and balance disorders [6]. Tactile sensitivity, joint movements, muscle strength, and vision all decrease with age; so does postural stability [7]. Improving postural stability in the elderly, therefore, seems crucial to reducing the likelihood of falls.

Postural stability is defined as the ability to control one’s body’s center of gravity within a given base of support [8]. Hence, as Hauer et al. showed, intensive balance and muscular strength training can lead to a reduction in falls compared to a control group [9].

Screening for risk of, and preventing, falls is not easy given the complexity of possible causes, but posturographic analysis is a promising way to identify individuals at risk of falling due to inadequate postural stability. Yet strategies to prevent falls often remain underutilized [10], because systems for measuring postural control are often expensive and time consuming (due to the complexity of analyzing test results) and, therefore, restricted to specialist institutions [11].

Home-based assessment and training would thus seem more ideal [9]. To this end, the Nintendo^®^ Wii Fit Balance Board (Nintendo, Kyoto, Japan), priced at around $100, might be an inexpensive and readily available tool. It is a sports-and-fitness device consisting of a force platform linked via Bluetooth to a video game console. Its integrated software, “Wii Fit,” contains 48 tasks divided into 4 groups: yoga, strength training, aerobics, and balance games.

By comparing the Wii Fit Balance Board to a laboratory-grade force platform, Clark et al. have already been able to prove its reliability and validity [12]. However, this was done by extracting raw data from the Wii rather than using the Wii Fit software’s integrated scoring system. Therefore, as a feasibility study, this project aimed at evaluating objectively the possibility of using the Wii Fit Balance Board as a postural stability diagnostic tool with its integrated scoring system, hypothesizing, that the results are comparable to the SOT, the gold standard in equilibrium analysis [13], as well as to certain other, well-established diagnostic and therapeutic devices, namely the MFT-S3 Check and Posturomed within a healthy population. To further evaluate the diagnostic relevance of the tools, we also checked for demographic differences among the study participants that may have affected the results.

## Methods

All volunteers provided oral and written consent to participate in this study and could discontinue at any time. The procedure for the study was approved by the RWTH Aachen ethics committee (EK 295/12) and conducted in accordance with the Declaration of Helsinki.

An orthopedic as well as an otorhinolaryngologic medical examination was conducted to confirm whether the subjects were capable of participating in this study, in which we included healthy adults (≥ 18 years of age). Exclusion criteria were: known deformities in or injuries to the spine, pelvis, or lower extremities; a pathological H.I.N.T.S (Head Impulse Test, Nystagmus und Test of Skew); incompliance; and underlying diseases attended by substantial restrictions such as diabetes or rheumatoid arthritis.

All subjects underwent testing with the Wii Fit Balance Board as well as with the Sensory Organization Test (SOT) (NeuroCom^®^, Pleasanton, USA), the MFT-S3 Check (TrendSportTrading GmbH, Großhöflein, Austria), and the Posturomed (Haider Bioswing GmbH, Pullenreuth, Germany).

**Fig. 1:**
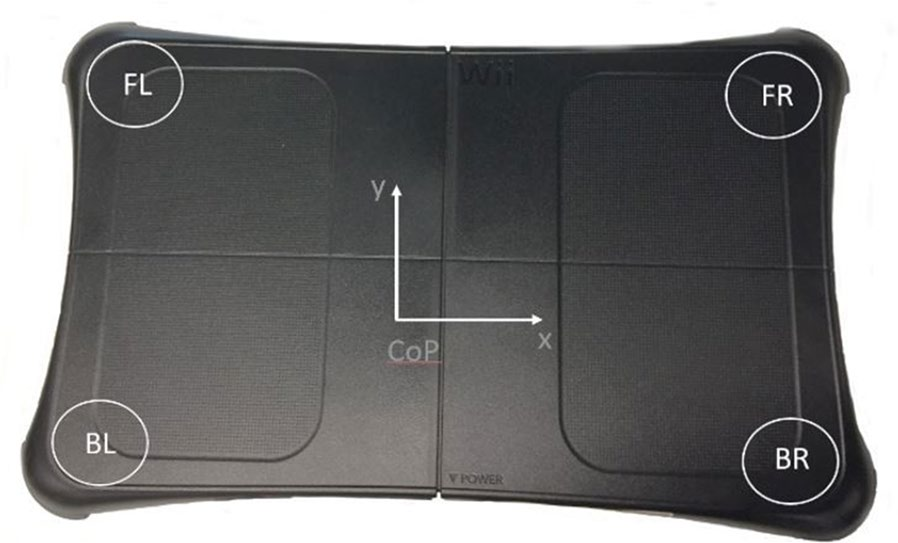
Force platform with the arrangement of pressure sensors

The Wii Fit Balance Board consists of a force platform with four pressure sensors (FL, FR, BL, and BR) which detect the force applied to the sensors as well as shifts in the center of balance through changes in the vector of the center of pressure (CoP), as depicted in Fig. 1.

**Fig. 2:**
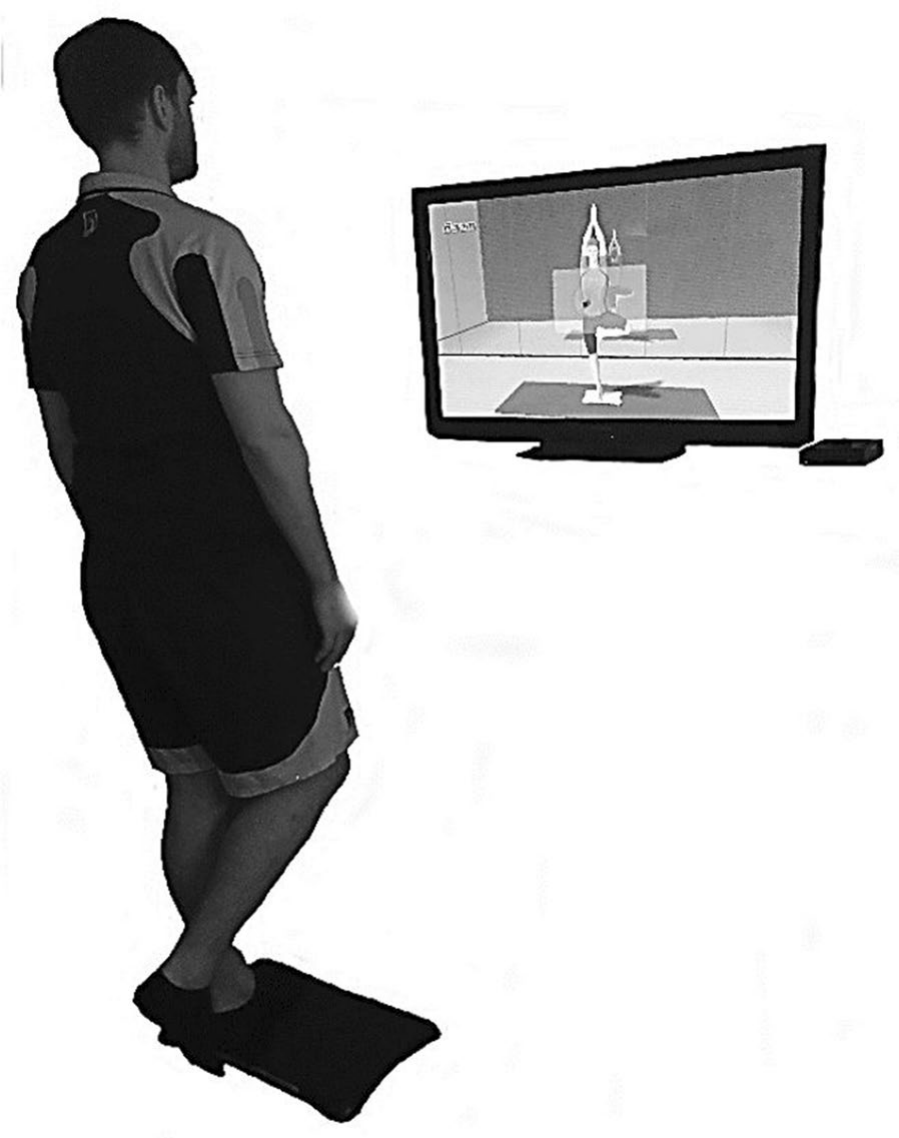
Set-up Yoga-task “tree” in one-leg-stance

**Fig. 3:**
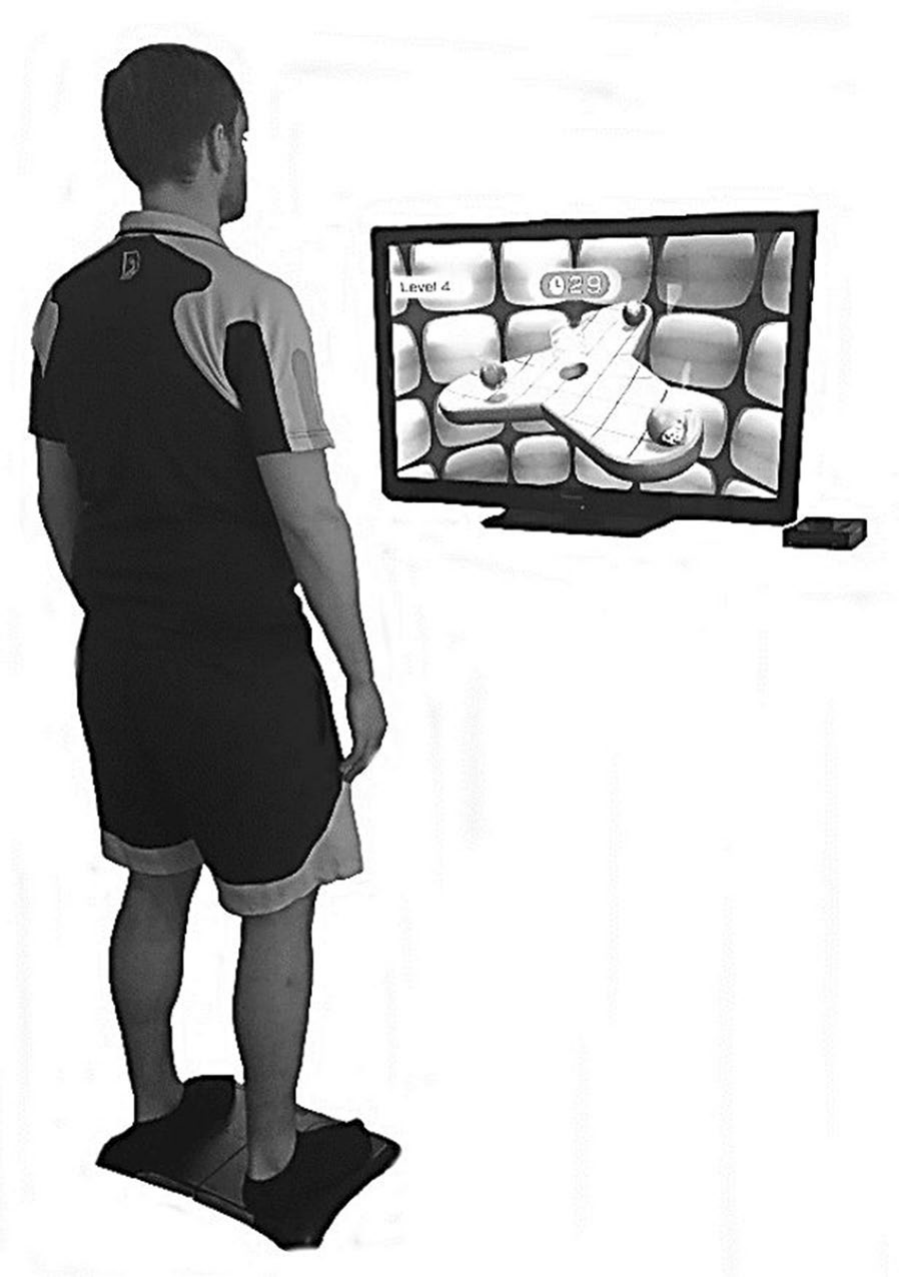
Set-up balance game “table-tilt”

We selected two of the integrated exercises for their high demands on postural stability, as well as for the similarity of testing compared to the MFT-S3 Check and the Posturomed. Our two selected exercises were the yoga-task “tree,” which calls for a one-legged stance, and the balance game “table tilt,” a task that requires great coordination in which the center of pressure must be shifted so as to roll a virtual ball into a pocket on a monitor. Figures 2, 3 show the setup, including the monitor used for visual feedback.

On the one hand, those two tasks differ in their respective aim: the yoga-task “tree” focuses on maintaining balance, whereas the subject intentionally transfers their center of pressure in the balance game “table tilt”. On the other hand, the scoring system also differs, as the yoga-task “tree” grades the result based on the deflection of the center of pressure (nominal value) and the balance game “table tilt” rewards points for accomplished levels (ordinal value).

The MFT-S3 Check utilizes an unstable platform connected to a base plate via the horizontal axis. This allows lateral pivoting of up to 12° to either side. The integrated sensors measure stability, sensory-motor regulatory capacity, and functional muscular asymmetry. By measuring the frequency and amplitude of sway, the system calculates a composite index for overall stability and sensory-motor capacity from 1 to 9, with 1 being the best score and 9 being the worst [13].

The Posturomed consists of an unstable platform suspended from four mountings and thus hanging in mid-air. Measurements are taken with the subject performing a one-legged stance on either leg. The amplitude and frequency of the movements of the platform are monitored, and a total score from 0 to 1000 is calculated [14].

The SOT utilizes six sensory conditions to evaluate a person’s standing balance. The SOT is administered with a computerized system using a movable dual forceplate and a movable visual screen. The SOT protocol assesses the patient’s ability to make effective use of visual, vestibular, and proprioceptive inputs as well as to suppress inaccurate sensory information. Differences in how much the patient’s body sways under the various sensory conditions determine their measured ability to organize and select the appropriate sensory information they need to maintain postural control [15]. The measurements result in an equilibrium score that ranges from 100 (no body sway) to 0 (fall) [16].

We conducted all measurements in the same order (SOT, Wii Fit, Posturomed, and MFT S3 Check), and all measurements were performed three times. A laboratory assistant supervised the subjects throughout the testing. The tests were performed with patients not wearing shoes and in a comfortable standing position with arms hanging down. The surroundings during testing were selected so as to be disturbance free.

### Statistical analysis

Statistical analysis was based on the recommendations of the local department for medical statistics. Given the different scoring systems used in the diagnostic tools under comparison, we converted the given value (*x*) by defining a maximum value (*a*) and a minimum value (*b*) for each measurement and generated a standardized value between “0” (worst score) and “1” (best score) using the following equation:

$$ {\text{Standardized value}}\, = \,\frac{x - b}{a - b}. $$

The three results for each diagnostic tool were averaged for statistical analysis, which was conducted with SPSS version 21 (SPSS inc., Chicago, USA) after testing for Gaussian distribution using the Kolmogorov–Smirnov test.

To analyze the relationship between the measurements, we used Pearson’s correlation. A correlation coefficient of 0.90–1.00 is sign of a very strong correlation; a coefficient of 0.70–0.89 represents a strong correlation; a coefficient of 0.4–0.69 represents a moderate correlation; 0.10–0.39 represents a weak correlation; and a coefficient of 0.00–0.09 represents a negligible correlation [17]. Furthermore, we conducted Bland–Altman plots to check for agreement as well as for possible bias.

To further characterize the diagnostic tools, we examined potential differences in demographic data. Nutritional status was measured using the body mass index (BMI) and participants were divided into the following groups according to the WHO [18]: catergory 1 (underweight) = BMI (kg/m^2^) < 19.9 (*n* = 7); category 2 (normal weight) = BMI 20.0–24.9 (*n* = 22); category 3 (overweight) = BMI 25.0–29.9 (*n* = 12).

Age was divided into groups as well (category 1: < 20 years (*n* = 9); category 2: 20–29 years (*n* = 23); category 3: 30–39 years (*n* = 3); category 4: 40–49 years (*n* = 2); category 5: > 50 years (*n* = 4).

Differences in the subjects’ demographic data were analyzed using paired t tests and ANOVAs.

The level of significance was set at *p* < 0.05.

## Results

A total of 41 subjects (21 females, 20 males) were examined in this study. The average age of the subjects was 27.9 years (SD ± 12.1), the youngest being 18 years of age, the oldest 70. They averaged 1.78 m (SD ± 0.11) and 73.5 kg (SD ± 17.1) in height and weight, thus averaging a BMI of 23.0 kg/m^2^ (SD ± 17.1).

**Fig. 4:**
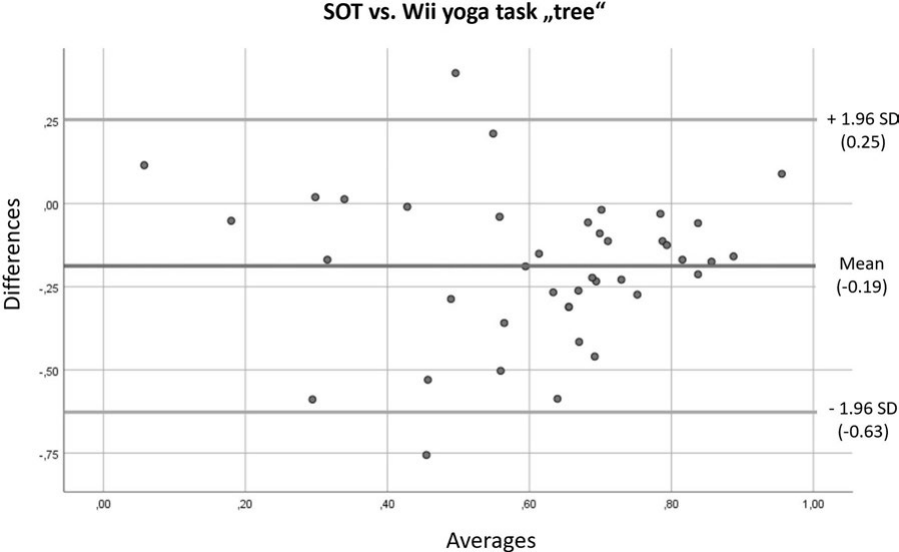
Bland–Altman plot for the SOT and the Nintendo Wii Yoga-task “tree”

**Fig. 5:**
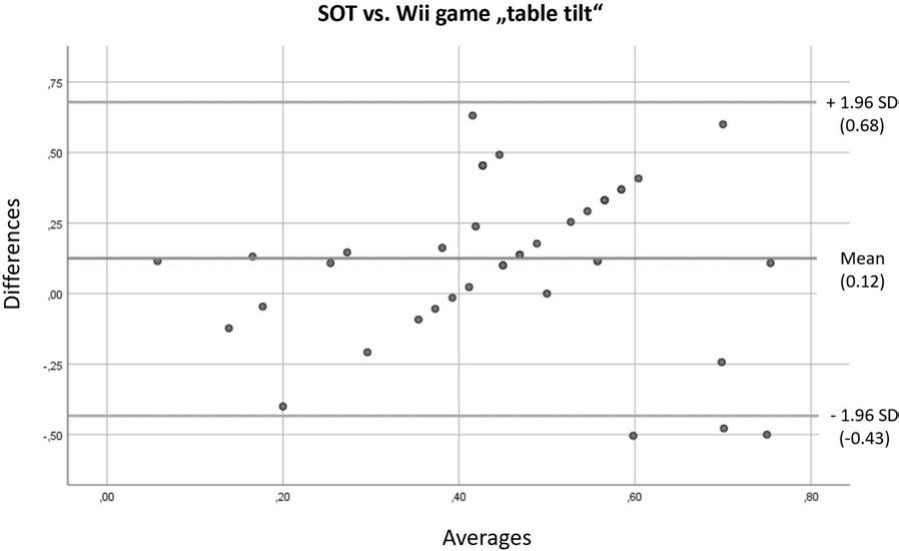
Bland–Altman plot for the SOT and the Nintendo Wii Fit game “table-tilt”

Analyzing the relationship between the scores on the Nintendo Wii and the test results from the established systems, we found considerable differences between the two selected tasks on the Wii Fit. Results for the yoga task “tree” significantly correlated with those of the SOT, doing so with a moderate correlation (*r* = 0.514; *p* = 0.001); however, there was no significant correlation between those of the SOT and the “table tilt” game (*p* = 0.301; *r* = 0.166). This difference also becomes evident when we view the Bland–Altman plots shown in Figs. 4, 5.

When we compare the Wii Fit’s “tree” yoga pose to the SOT, the Bland–Altman plot shows a certain bias, and furthermore a trend, as the difference between the results decreases with higher mean values. When we compare the Wii Fit’s “table tilt” game with the SOT, we see a bias as well as a linear relationship between the means and the difference; this shows that both results differ significantly.

Analyzing the relationships between the SOT (as the gold standard) and each of the established systems, we also find a significant correlation for the results of the MFT-S3 Check (sensorimotor index: *r* = 0.404; *p* = 0.009, and stability index: *r* = 0.347; *p* = 0.011). For the Posturomed, on the other hand, no significant correlation with the SOT could be detected (*p* = 0.242).

Examining the relationships between the Nintendo Wii Fit and both the MFT-S3 Check as well as the Posturomed, we found a significant correlation between the yoga task “tree” and both of the other measurement tools as shown in Table 1:

**Table 1:** Correlation coefficient with established systems compared to Wii Fit yoga-task “tree”

	Correlation coefficient	Level of significance
Posturomed	0.345	0.027
MFT-S3 Check sensorimotor	0.356	0.022
MFT-S3 Check stability	0.472	0.002

**Fig. 6:**
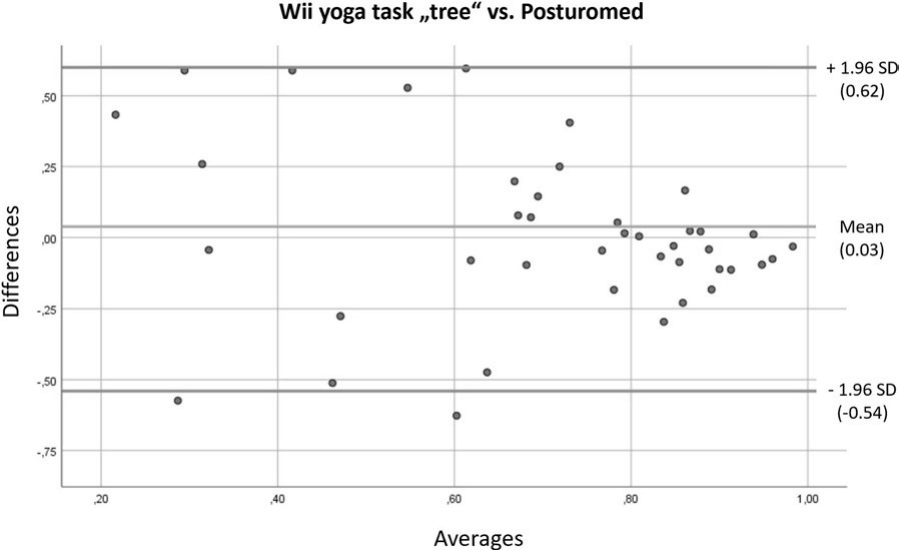
Bland–Altman plot for the Nintendo Yoga-task “tree” and the Posturomed

**Fig. 7:**
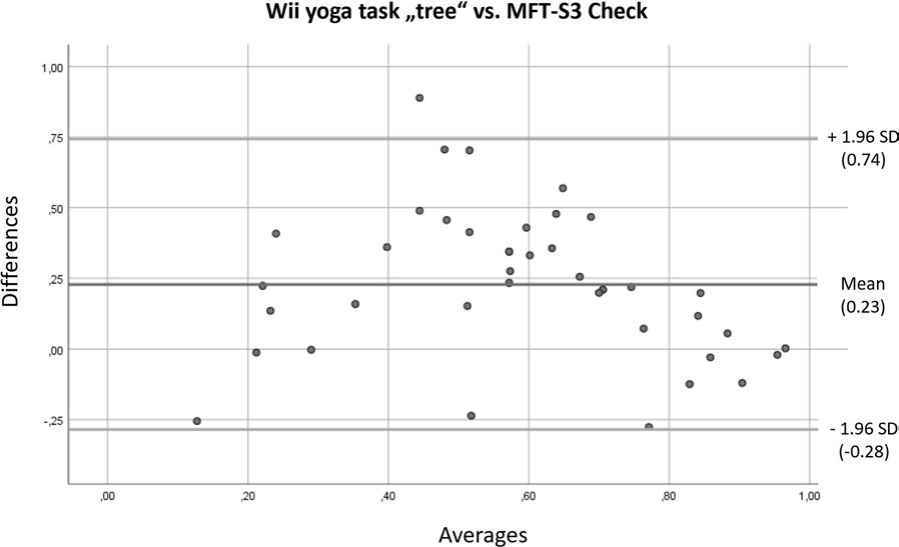
Bland–Altman plot for the Nintendo Yoga-task “tree” and the MFT-S3 Check

Figures 6, 7 show Bland–Altman plots for the comparisons of the Wii Fit’s “tree” yoga pose to the Posturomed and the MFT-S3 Check`s stability-result, respectively.

A comparison of the results of the Wii Fit’s “table tilt” game with those of the established systems revealed no significant correlation (see Table 2).

**Table 2:** Correlation coefficient with established systems compared to Wii Fit “table tilt” game

	Correlation coefficient	Level of significance
Posturomed	0.142	0.375
MFT-S3 Check sensorimotor	− 0.054	0.736
MFT-S3 Check stability	0.10	0.953

Furthermore, we assessed gender and demographic differences for each measurement tool.

As far as gender differences are concerned, we found no significant differences for either Wii Fit Balance Board measurement (*p* = 0.0368, *p* = 0.99); nor did we find significant differences for the SOT and Posturomed (*p* = 0.781 and *p* = 0.559, respectively). The MFT-S3 Check was the only system for which a difference was found, with women scoring significantly higher (*p* = 0.004), with a mean value of 0.502 (SD 0.264), than men did, with a mean value of 0.355 (SD 0.236).

As far as nutritional status is concerned, the MFT-S3 Check was likewise the only diagnostic device to show significant differences (*p* = 0.001).

Bonferroni post hoc testing found a significantly higher score for the underweight category 1 group, with a mean value of 0.808 (SD 0.179), than there was for the other groups (*p* = 0.001–0.004). Directly comparing groups 2 (mean value 0.458; SD 0.249) and 3 (mean value 0.332; SD 0.209) revealed no significant difference (*p* = 0.246). However, there was a significant (*p* = 0.000) negative correlation between the results of the MFT-S3 Check and BMI (*r* = − 0.529).

The results of the SOT (*p* = 0.4), Wii Fit Balance Board (*p* = 0.128; *p* = 0.320), and Posturomed (*p* = 0.939) showed no significant differences as to nutritional status.

As far as the age of the subjects is concerned, only the yoga-task “tree” on the Nintendo Wii showed significant differences between the various age groups (*p* = 0.017). Post hoc analysis detected significant differences between age groups 1 (mean 0.694; SD 0.191) and 5 (mean 0.393; SD 0.239) *p* = 0.020; between groups 2 (mean 0.746; SD 0.210) and 5 (*p* = 0.031); and between groups 3 (mean 0.915; SD 0.072) and 5 (*p* = 0.021). Pearson’s correlation detected a significant, yet weak negative correlation between the results and the age of the subjects, of r = − 0.35 (*p* = 0.025).

The “table tilt” balance game on the Nintendo Wii did not reveal any significant difference between the age groups (*p* = 0.387). The established systems were likewise unable to measure significant differences in postural stability among the age groups (SOT: *p* = 0.571; MFT-S3 Check: *p* = 0.883; Posturomed: *p* = 0.641).

## Discussion

In this study of 41 healthy subjects, we showed that the Wii Fit yoga-task “tree” significantly correlates with the established system on direct comparison. There is a weak-to-moderate correlation between all systems and the Wii Fit yoga-task “tree” (*r* = 0.345–0.514). The reason for the correlation being not greater than weak to moderate could be that even though all devices aim at identifying balance or imbalance, they all have different approaches to measuring it. Furthermore, the scoring system differs between the devices. To properly analyze and compare the measurements, a general score has been calculated, which might lead to a bias.

The Bland–Altman plots also showed that the methods generally produce different results. But there is an obvious relationship between the respective results nonetheless, and the difference between the results decreases when subjects score highly. This effect can be seen upon comparing the yoga-task “tree” on Wii Fit with the SOT as well as the Posturomed. We, therefore, conclude that the measurement method in combination with the software underlying the yoga-task “tree” can be considered an adequate diagnostic tool.

The results for the Nintendo Wii Fit’s game “table tilt,” however, do not correlate with the other measurement tools at all. The Bland–Altman plot furthermore showed an obvious trend, with a substantial bias; and “table tilt” should therefore not be considered adequate as a diagnostic device.

When it comes to demographic differentiations, the results of the Nintendo Wii Fit Balance Board should comply with literature. Here it is stated that the age adversely affects postural stability [19], which is in line with the results of the “tree” pose on Wii Fit, especially showing, that the age group > 50 years scores significantly lower. But the Wii Fit was unable to differentiate between genders or various nutritional states despite the statement by Hue et al. that decreasing postural stability is strongly correlated to increasing body weight [20]. As far as gender differences are concerned, several studies have been unable to identify a significant difference in postural stability [21–23].

Regarding the established systems both SOT and Posturomed could not detect differences regarding gender, age, or nutritional status. Only the MFT-S3 Check was able to detect differences in the nutritional status as well as in gender with women scoring significantly higher.

Since postural instability is a risk factor for falls, especially in the population aged 65 years of age or older [24, 25], screening for instability is an important step toward preventing the falls associated with fragility fractures. Therefore, the detection of postural instability can either be used as a tool for secondary or tertiary prevention, but can also be used for primary prevention, for example by a general physician. To achieve that, measurement tools should be widely achievable and cost-efficient, which is the case with the Nintendo Wii Fit Balance Board.

Furthermore, studies have shown that proprioceptive training is effective in older adults at improving postural stability, static and dynamic balance, helping lower the risk of suffering a fall [26]. Additionally, a systematic review has found higher adherence to technology-based programs, such as the Wii Fit, compared to regular exercise programs [27], when it comes to continuing a training program.

Numerous studies have indicated that the Wii Fit Balance Board could be used to treat the imbalance symptoms of diseases such as Parkinson’s, cerebral palsy, stroke, multiple sclerosis, or vestibular disorders [28, 29]. Further applications of this system include rehabilitation after injury to or surgery on the lower extremities as well as injury prevention in athletes [30–32].

This shows that the Nintendo^®^ Wii Fit Balance Board can be used not only as a diagnostic but also as a therapeutic device. This sets the Wii apart from many other tools, for example the Sensory Organization Test.

Examining the Wii Fit Balance Board, Chang et al. furthermore showed that it provides highly reliable measurements in the elderly as well as a good intra-class correlation and a high correlation with the results of the Balance Master System^®^ (NeuroCom^®^, Pleasanton, USA). For their study, however, they obtained and analyzed raw data from the Wii Fit Balance Board using software they designed themselves [33]. Unlike our study, in which we make use of software already integrated into the Nintendo^®^, that approach contains a limitation on the usefulness of the Wii Fit Balance Board because of the technical difficulty of extracting raw data from the Wii, and because of the question of making a software widely available for analyzing the raw data.

But since this is the first study providing evidence for the use of Nintendo`s integrated software and scoring system as a tool for assessing postural stability, the limitations of our study must be considered.

The Nintendo Wii Fit Balance Board can be considered a portable and low-cost posturography system to monitor improvements or a decline in postural stability of one individual. But, further studies are necessary to determine the normative values as well as a cut-off value to detect people with an increased risk of falling.

Furthermore, we primarily studied young and healthy individuals; and thus, further studies must evaluate the practicability of this technique among a geriatric patient population. While all measurement tools are designed to evaluate postural stability and the sense of equilibrium, each comes with its own approach to measurement, which impedes any direct comparison.

## Conclusions

With this study, we have been able to show that the Wii Fit Balance Board, specifically its yoga-task “tree”, achieves differentiated results as compared to more established systems and can, therefore, be used as a portable, low-cost posturography system. However, normative values for the Nintendo Wii Fit Balance Board have yet to be determined when it comes to assessing patients’ risk of suffering a fall. Further studies are needed to address this issue.

## Data Availability

The datasets used and/or analyzed during the current study are available from the corresponding author on reasonable request.

## Abbreviations

SOT: Sensory organization test
SD: Standard deviation
m: Meter
kg: Kilogram
H.I.N.T.S: Head impulse test, nystagmus und test of skew
FL: Front left
FR: Front right
BL: Back left
BR: Back right
CoP: Center of pressure
a.p.: Anteroposterior
BMI: Body mass index
WHO: World Health Organization
p: Level of significance
Std.: Standard
